# The Amyloid-β Pathway in Alzheimer’s Disease

**DOI:** 10.1038/s41380-021-01249-0

**Published:** 2021-08-30

**Authors:** Harald Hampel, John Hardy, Kaj Blennow, Christopher Chen, George Perry, Seung Hyun Kim, Victor L. Villemagne, Paul Aisen, Michele Vendruscolo, Takeshi Iwatsubo, Colin L. Masters, Min Cho, Lars Lannfelt, Jeffrey L. Cummings, Andrea Vergallo

**Affiliations:** 1 Eisai Inc., Neurology Business Group, Woodcliff Lake, NJ USA; 2 UK Dementia Research Institute at UCL and Department of Neurodegenerative Disease, UCL Institute of Neurology, University College London, London, UK; 3 Clinical Neurochemistry Laboratory, Sahlgrenska University Hospital, Mölndal, Sweden; 4 Institute of Neuroscience and Physiology, Department of Psychiatry and Neurochemistry, the Sahlgrenska Academy at the University of Gothenburg, Mölndal, Sweden; 5 Memory Aging and Cognition Centre, Departments of Pharmacology and Psychological Medicine, Yong Loo Lin School of Medicine, National University of Singapore, Singapore, Singapore; 6 Department of Biology and Neurosciences Institute, University of Texas at San Antonio (UTSA), San Antonio, TX USA; 7 Department of Neurology, College of Medicine, Hanyang University, Seoul, Republic of Korea; Cell Therapy Center, Hanyang University Hospital, Seoul, Republic of Korea; 8 Department of Psychiatry, University of Pittsburgh, Pittsburgh, PA USA; 9 Department of Medicine, The University of Melbourne, Melbourne, VIC Australia; 10 USC Alzheimer’s Therapeutic Research Institute, San Diego, CA USA; 11 Centre for Misfolding Diseases, Department of Chemistry, University of Cambridge, Cambridge, UK; 12 Department of Neuropathology, Graduate School of Medicine, The University of Tokyo, Tokyo, Japan; 13 Laureate Professor of Dementia Research, Florey Institute and The University of Melbourne, Parkville, VIC Australia; 14 Uppsala University, Department of of Public Health/Geriatrics, Uppsala, Sweden; 15 BioArctic AB, Stockholm, Sweden; 16 Chambers-Grundy Center for Transformative Neuroscience, Department of Brain Health, School of Integrated Health Sciences, University of Nevada Las Vegas (UNLV), Las Vegas, NV USA

**Keywords:** Neuroscience, Diseases, Diagnostic markers

## Abstract

Breakthroughs in molecular medicine have positioned the amyloid-β (Aβ) pathway at the center of Alzheimer’s disease (AD) pathophysiology. While the detailed molecular mechanisms of the pathway and the spatial-temporal dynamics leading to synaptic failure, neurodegeneration, and clinical onset are still under intense investigation, the established biochemical alterations of the Aβ cycle remain the core biological hallmark of AD and are promising targets for the development of disease-modifying therapies. Here, we systematically review and update the vast state-of-the-art literature of Aβ science with evidence from basic research studies to human genetic and multi-modal biomarker investigations, which supports a crucial role of Aβ pathway dyshomeostasis in AD pathophysiological dynamics. We discuss the evidence highlighting a differentiated interaction of distinct Aβ species with other AD-related biological mechanisms, such as tau-mediated, neuroimmune and inflammatory changes, as well as a neurochemical imbalance. Through the lens of the latest development of multimodal in vivo biomarkers of AD, this cross-disciplinary review examines the compelling hypothesis- and data-driven rationale for Aβ-targeting therapeutic strategies in development for the early treatment of AD.

## Introduction

Alzheimer’s disease (AD) is the primary cause of dementia, affecting ~45.0 million individuals worldwide and is ranked as the fifth leading cause of death globally [1]. In the United States alone, an estimated 5.8 million individuals live with AD dementia today, and this number is expected to grow to 13.8 million by 2050 [2, 3]. Similarly, in Western Europe, dementia affects ~2.5% of people aged 65–69 years, escalating to about 40% of those aged 90–94 years [4], and by 2050, there will likely be up to 18.9 million patients with dementia in Europe [5] and 36.5 million in East Asian countries [1].

To date, drugs approved for the treatment of AD are labeled for the disease’s clinical dementia stage and target the neurochemical systems underlying cognitive dysfunction and behavioral symptoms, with only short-term symptomatic effects. In the last 25 years, translational studies—including experimental animal and human neuropathological, genetic, and in vivo biomarker-based evidence—support a descriptive hypothetical model of AD pathophysiology characterized by the upstream brain accumulation of Aβ species and plaques, which precedes spreading of tau, neuronal loss and ultimately clinical manifestations by up to 20–30 years [6]. Such multi-dimensional evidence led to reshaping the conceptual framework of AD, into a clinical-biological construct along a continuum that spans preclinical, prodromal, and eventually dementia stages [6, 7].

This pathophysiological model has supported a considerable effort to develop therapeutic compounds targeting the Aβ pathway to slow AD progression in early clinical stages. More recently, several anti-Aβ therapeutic pipelines have been expanded to preclinical stages of AD, when the expected success rate of compounds with putative biological effects is higher [8]. While research and physician communities have raised theoretical and conceptual questions on the scientific appeal of Aβ-targeting therapeutic development due to the failures of AD drug clinical trials, anti-Aβ compounds are continually investigated with promising progress of several late-stage development agents towards regulatory approval steps. Moreover, thorough evaluation of disease relevance of a biological pathway—including sophisticated incorporation of latest biomarkers for target engagement, optimized dosing, and selection of participants and treatment response monitoring despite highly heterogenous populations and subsequent results—may help dispel the concern that negative clinical trials negate the true biological and pathophysiological validity of a complex entity such as the Aβ pathway in AD. Critical evaluation of the Aβ pathway in the sole context of clinical trials is a worthy topic for discussion and have been discussed frequently. Critical evaluation of evidence independent of clinical trial results of anti-Aβ drugs can provide the rationale and validation of the disease relevance of the Aβ pathway, especially as data from supporting non-clinical studies of the Aβ pathway continue to accrue.

In this evolving landscape, we present a systematic and cross-disciplinary state-of-art update of the translational literature based on genetic, epigenetic, and biological data that support the pathophysiological role of the Aβ pathway in the biological continuum of AD. We deliver a descriptive evidence-based overview without inferring any causal nexus between the Aβ pathophysiology and other established AD-related pathophysiological alterations occurring at different temporal scales. This multi-perspective endeavor describes an evidence-based state-of-the-art of the literature that points out a rationale for Aβ-targeting therapeutic strategies for the early treatment of AD and identifies knowledge gaps.

## Early History of the Amyloid-β Pathway in AD

The Aβ is a 4 kDa fragment of the amyloid precursor protein (APP), a larger precursor molecule widely produced by brain neurons, vascular and blood cells (including platelets), and, to a lesser extent, astrocytes. Two subsequent proteolytic cleavages of APP by β-secretase (β-APP-cleaving enzyme-1 (BACE1)) at the ectodomain and γ-secretase at intra-membranous sites generate Aβ [9]. In 1984, Aβ and its amino acid sequence were reported for the first time as a primary constituent of meningovascular polymorphic deposits in patients with Down Syndrome; the full sequence of parenchymal Aβ plaque core was found to be identical to the peri-vascular component previously described except that the latter mainly extends to the 42nd residue [10]. Subsequently, the *APP* gene was sequenced, corroborating that Aβ is a by-product of the enzymatic processing of APP [11]. Eventually, dense Aβ aggregates were described as the main constituent of neocortical neuritic plaques, characterizing brain aging and constituting a pathological hallmark of AD along with tau neurofibrillary tangles (NTFs) [12].

**Fig. 1:**
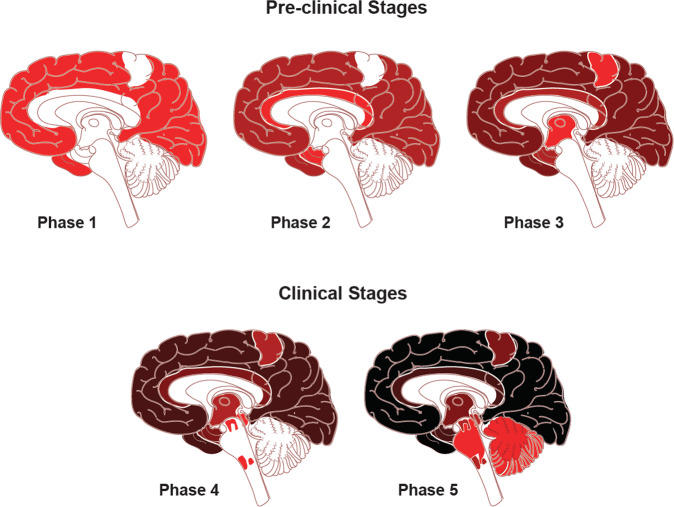
Traditional neuropathological phases of amyloid-β deposition in Alzheimer’s disease dementia. Red areas in Phase 1 depicts the cortical regions with the initial accumulation of amyloid-β during the early pre-clinical stage. Continued deposition in the same areas are shown in darker colors in the subsequent stages, with the new areas showing amyloid-β in red in each phase. Neocortical regions with the early accumulation of amyloid-β in phase 1 include association cortices. Additional accumulation is seen in allocortical regions and midbrain (phases 2 and 3), with the cerebellum and brain stem having amyloid-β accumulation in late phase clinical stages. The change to darker shading indicates the continuous accumulation of Aβ. Adapted with permission from ref. [13].

**Fig. 2:**
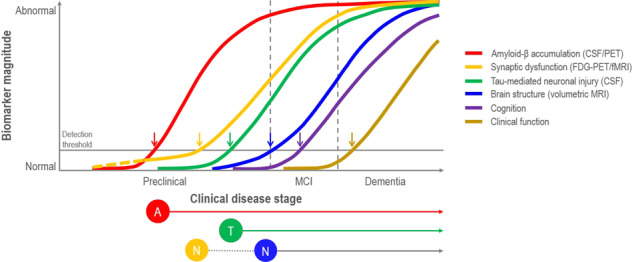
Hypothetical biomarker evidence-driven model of AD pathophysiology. Hypothetical model of dynamic biomarkers of the AD is expanded to explicate the preclinical phase. Aβ is identified by cerebrospinal fluid Aβ42 assay or PET amyloid imaging. Synaptic dysfunction evidenced by [^18^F]-fluorodeoxyglucose positron emission tomography (FDG-PET) or functional magnetic resonance imaging (fMRI), with a dashed yellow line to indicate that synaptic dysfunction may be detectable in carriers of the ε4 allele of the apolipoprotein E gene before detectable Aβ deposition. Neuronal injury is evidenced by cerebrospinal fluid tau or phospho-tau, and brain structure is documented by structural magnetic resonance imaging. Biomarkers change from normal to maximally abnormal (*y*-axis) as a function of disease stage (*x*-axis). The temporal trajectory of two key indicators used to stage the disease clinically, cognitive and behavioral measures, and clinical function are also illustrated. Neurofilament light chain (NfL) and neurogranin are newer and potentially more accurate markers of neuronal injury. Figure adapted with permission from ref. [391].

Neuropathological studies, confirmed in vivo by recent quantitative neuroimaging investigations, indicate a spatial-temporal evolution of brain Aβ accumulation that occurs initially in cerebral regions with neuronal populations at high metabolic bio-energetic activity rates (such as association cortices) and spreads from neocortex to allocortex to brainstem, eventually reaching the cerebellum (see Fig. 1) [13]. During the 1990’s and early 2000’s, (i) mechanistic studies linking autosomal dominant AD genes, (ii) investigation of several genetic risk factors relating late‐onset AD to Aβ accumulation, and (iii) longitudinal biomarker-based studies conducted in individuals at risk led to draw the biological-clinical construct for AD including the evidence that Aβ pathophysiology occurs decades before the onset of clinical symptoms [14–16]. In addition, brain Aβ accumulation appears to be upstream to other pathomechanistic alterations of the biological continuum of AD, including the spreading of NTFs, and involvement of neuronal and synaptic loss (Fig. 2). The temporal and spatial evolution of these pathophysiological alterations underlies AD cognitive and functional decline across a clinical continuum, from preclinical to prodromal and dementia stages.

Experimental pathomechanistic and proof-of-concept studies indicate an imbalance between Aβ neuronal production and extracellular clearance of Aβ as the upstream event of Aβ dyshomeostasis, associated with protein misfolding, aggregation, and incipient extracellular accumulation in plaques [15, 17, 18]. While in early-onset AD (EOAD) such an imbalance is primarily due to genetic-driven deregulation of the amyloidogenic pathway with downstream overproduction of Aβ, in late‐onset cases of AD (LOAD) failure of proteostasis networks—mechanisms quality control, from protein synthesis to protein degradation—with insufficient cerebral Aβ clearance represents the key event in Aβ aggregation [19]. Such trickle-down effects comprise the initiating factor of brain Aβ accumulation as an early and central pathophysiological alteration within the AD biological continuum [7, 15, 17].

## Genetic Evidence of the Role of the Aβ Pathway

### Early-onset AD (EOAD)

Large-scale genetic analyses conducted in datasets of informative monogenic EOAD pedigrees identified highly penetrant mutations in the three genes—the APP gene and the presenilin 1 and 2 (*PSEN1* and *PSEN2*) genes. These mutations are transmitted through autosomal dominant inheritance (i.e., autosomal dominant Alzheimer’s disease or ADAD). In mouse models of ADAD each monogenic mutation causes Aβ dyshomeostasis, with protein misfolding, aggregation, and accumulation in brain parenchymal Aβ plaques [15, 17, 20–22]. Such a linear pathomechanistic model (i.e., “one mutation-one misfolded protein”) led to the conceptualization of the “amyloid cascade” [20–22]. In humans, genetic EOAD accounts for around 1% of all AD cases, and most of the genetic forms are caused by mutations in the *APP*, *PSEN1*, and *PSEN2* genes, with more than 300 different autosomal dominant mutations reported in these genes [23, 24].

The locus of the APP gene is on chromosome 21. Several genetic linkage studies and observational data indicate that individuals with Down syndrome, bearing APP gene triplication, develop cognitive impairment associated with AD biological signatures [25, 26]. Moreover, 25 genomic duplications encompassing *APP* were found to co-segregate with AD in families with autosomal dominant disease transmission [27, 28]. Most pathogenic mutations on the *APP* gene cluster around the proteolytic sites of the β- and γ-secretases with a downstream increase of the substrate affinity and either an overall increase of the total Aβ pool or shifts in Aβ peptides ratios. The latter is characterized by a relative increase of Aβ1-42 levels over the levels of Aβ1-40 and shorter species [25–28]. Such an imbalance is hypothesized to facilitate protein self-aggregation [29, 30].

The potential pathogenic role of the APP gene in humans is supported by the existence of a rare protective variant—*APP A673T* (or *A2T*)—next to the APP β-secretase site that reduces both APP cleavage and the production of amyloidogenic Aβ peptides [25–28]. The *A673T* rare variant is five times more common in non-demented older Icelandic individuals than in AD [31]. Notably, another novel variant of this gene—*A673V*—is linked to AD when the individual is homozygous for the gene, whereas the heterozygous state is unaffected, in line with a model of recessive Mendelian trait type of inheritance [32]. The opposite effects of *APP A673V* and *APP A673T* variants on amyloidogenesis indicate a distinct autosomal recessive pattern of inheritance [33]. *PSEN1* accounts for most of the known AD-related mutations with the autosomal dominant transmission. Over 200 mutations involving this complex have been observed [34]. *PSEN2* mutations are rare, with less than 40 mutations currently identified [31, 35]. In vitro studies and PSEN1/PSEN2 gene knockout mouse models show (i) reduced NOTCH signal due to a diminished cleavage, (ii) decreased formation of the APP Intracellular Domain fragment (AICD), and (iii) reduced processing of other γ-secretase substrates (see below for more information about the PSEN complex biology). These studies point at a genetically-driven γ-secretase loss of function [36]. Several pathogenic *PSEN1/2* mutations induce a unique partial loss of function of APP γ-secretase-dependent cleavage, associated with a shift to Aβ1-42 position cleavage, and decrease both Aβ1-42 and Aβ1-40 production [37]. Unlike *PSEN1*, AD patients carrying *PSEN2* mutations exhibit a wide range of age of onset, from 40 to 80 years. Mutations in PSEN2 have been reported in association with other diseases, including frontotemporal dementia (FTD), dementia with Lewy bodies (DLB), breast cancer, and dilated cardiomyopathy [38].

### Late-onset AD (LOAD)

At present, no causal (autosomal dominant or recessive) genetic mutations are known in association with late-onset AD [39]. LOAD is hypothesized to be a multifactorial disease with a complex genetic background. Several critical genetic risk factors in AD susceptibility have been detected through large-scale genome-wide association studies (GWASes), with more than 50 susceptibility genes/loci associated with LOAD risk(see Table 1) [39]. Although GWASes do not uncover causative mechanisms, it is notable that many of these genes are linked to Aβ homeostasis, including its (i) expression (*APP, PSEN1, PSEN2* and *ADAM10*), (ii) trafficking (*APOE, CLU* and *SORL1*), and (iii) degradation (*PICALM, SORL1, CD33, BIN1, CD2AP, ABCA7*, and *RIN3* are associated with the endosomal-lysosomal system, and *CLU* and *PTK2B* are associated with the ubiquitin-proteasome pathway) [40].

**Table 1:** Loci reaching genome-wide significance for association with sporadic late-onset AD.

Locus	GWS locus or gene	original SNP and publication	Dataset	Functional information
1	*APOE*	rs429358 p.(Cys112Arg); ref. [380].	Case–control	A multifactorial protein, known primarily for its role in lipid transport. Known to bind soluble Aβ.
		rs7412 p.(Cys158Arg); ref. [380].		
3	*CLU*	rs11136000; refs. [381, 382].	GERAD EADI	Molecular chaperone. Role in immunity and cholesterol metabolism. Binds Aβ.
7	*TREM2*	rs75932628 p.(Arg47His); refs._._ [383, 384]	Mixed-cohorts	Receptor of the immunoglobulin superfamily, binds lipids and Aβ. Signals to affect multiple processes in myeloid cells including phagocytosis and cellular metabolism.
		rs143332484 p.(Arg62His); ref. [385].	IGAP	
15	*BIN1*	rs744373; ref. [386].	CHARGE	Involved in endocytic recycling and Aβ production. also involved in membrane folding.
21	*SORL1*	rs11218343; ref. [40].	IGAP	Endocytic receptor involved in the uptake of lipo- proteins, APP processing and lysosomal targeting of Aβ.
		Gene-wide; ref. [387]	ADES-FR	
22	*ABCA7*	rs3764650; ref. [388].	GERAD+	Transporter involved in cholesterol metabolism and phagocytic clearance of Aβ.
		Gene-wide; ref. [389]	IGAP	
25	*ADAM10*	rs593742; refs. [383, 390].	IGAP+	Metalloprotease responsible for proteolytic processing of APP.
			Combined UK Biobank	
			and IGAP	
36	*APP*	rs63750847, p.(Ala673Thr); ref. [107].	Icelandic, Finnish	APP.
			and Swedish	
37	*IGHG3*	rs77307099; ref. [384].	ADSP	Immunoglobulin gene whose antibodies interact with Aβ.

Datasets: Alzheimer’s disease sequencing project (ADSP); Psychiatric Genomics Consortium Alzheimer’s disease working group (PGC–ALZ); deCODE, a private corporation (https://www.decode.com); Genetic and Environmental Risk in AD (GERAD); International Genomics of Alzheimer’s Disease Consortium (IGAP); European AD Initiative (EADI); Cohorts for Heart and Aging Research in Genomic Epidemiology (CHARGE); Alzheimer’s Disease Exome Sequencing-France (ADES-FR).

Aβ amyloid beta, *APP* amyloid precursor protein. Table adapted from ref. [39].

In addition, pathway analyses indicate that polymorphisms in these genes may have a pleiotropic effect or may not be directly linked to the Aβ pathway but encode for proteins whose alterations are associated with impairment of Aβ homeostasis with a network-wise effect. Several genes related to LOAD play a role in the regulation of inflammatory and immune response pathways, endocytosis and cellular trafficking, cholesterol transport and lipid metabolism, post-translational modification—including ubiquitination, which is a crucial mechanism of cellular protein clearance; see Table 1 for details [39].

### The association between APOE ε4 and the Aβ pathway

The apolipoprotein E (APOE) ε4 allele (locus on chromosome 19q13.2) is the first and most significant LOAD risk gene identified [41, 42]. A significant detrimental effect of APOE ε4 allele on EOAD pathophysiology has also been reported [43]. Age-related memory trajectories in APOE ε4 carriers may diverge from those of non-carriers before the age of 60 years despite ongoing normal clinical status as the presence of APOE ε4 correlates with an earlier decline [44]. Homozygosity for the APOE ε4 allele increases the risk of developing LOAD by 3- to 15-fold in a dose-dependent manner [45]. *APOE* has three major allelic variants, *APOE ε2*, *APOE ε3*, and *APOE ε4*, with the ε3 allele being the most common (77%) and ε2 allele the least common (8%) [46]. Human ApoE protein is a 34-kDa glycoprotein consisting of 299 amino acids. In the central nervous system (CNS), ApoE is abundantly expressed in astrocytes, microglia, vascular mural cells, and choroid plexus cells, and, to a lesser extent, in stressed neurons [45]. ApoE isoforms differentially modulate multiple brain intracellular signaling pathways, including lipid transport, synaptic homeostasis, glucose metabolism, and cerebrovascular function [45].

Clinical and neuropathological studies show a significant association between *APOE* genotype and Aβ metabolism and homeostasis [45, 47–49]. Brain tissue from AD patients shows that *APOE ε4* is correlated with increased intraneuronal accumulation of misfolded Aβ, formation of neurotoxic Aβ species, and plaque parenchymal accumulation [45, 47–49]. Both neuroimaging and cerebrospinal fluid (CSF) biomarker studies indicate a consistent association of *APOE ε4* with higher cerebral Aβ deposition in cognitively healthy elderly individuals and across the full clinical continuum of AD, i.e., in patients with subjective memory complaint, prodromal (or mild cognitive impairment (MCI)) and dementia [50–54].

The *APOE ε4* effect is marked by earlier AD symptoms onset in cognitively healthy individuals with positive Aβ biomarkers [55] but with otherwise typical clinical progression. The impact of the *APOE* genotype on the risk of AD cognitive-functional decline is likely to be Aβ-mediated [56]. The effect of *APOE ε4* on Aβ metabolism and aggregation appears to be most pronounced during the initiation phase of Aβ dyshomeostasis [57]. Increasing age exacerbates this effect, indicating a potential synergistic interaction between *APOE* and aging-related metabolic changes [58]. Investigation of the combined *APOE ε4*-age effect on Aβ accumulation has gained traction since it may help develop reliable predictive models of AD clinical trajectories in cognitively healthy at-risk individuals [45].

### The link between the APOE ε4 allele and brain Aβ accumulation: experimental evidence

Studies in humans and transgenic mice support that a model in which brain levels of Aβ species aggregation and rates of Aβ plaque formation are ApoE isoform-dependent (ε4>ε3>ε2), allowing inference of a role for ApoE in modulating Aβ metabolism, aggregation, and deposition [45, 59]. Although the molecular dynamics underlying a direct effect of ApoE isoforms on amyloidogenic pathways are not elucidated yet, studies in vitro and in mouse models of AD indicate that ApoE modulates γ‑secretase activity and downstream Aβ production [60, 61].

ApoE upregulates *APP* transcription and Aβ production in human embryonic stem cells-derived and induced pluripotent stem cell (iPSC)-derived neurons in an isoform-dependent fashion (i.e., ApoE4 stimulating Aβ production more effectively than ApoE2 or ApoE3) [62]. Furthermore, Aβ secretion was significantly higher in iPSC-derived neurons carrying *APOE ε4* than in those with *APOE ε3*, probably due to increased APP transcription or splicing [63, 64].

Preliminary in vivo evidence indicates that APP processing is not affected by ApoE isoforms [65]. By contrast, mouse models show that a primary mechanism for ApoE-mediated plaque formation to be effects of ApoE on aggregation dynamics rather than from isoforms themselves [66]. Some studies indicate that ApoE4 can facilitate the formation of Aβ fibrils by accelerating the initial seeding or nucleation of Aβ deposition [45, 67]. Astrocytic overexpression of ApoE4—but not ApoE3—was found to exacerbate Aβ seeding and increase brain Aβ half-life in a mouse model of aging [45, 67]. ApoE4 expression increased, whereas ApoE3 reduced, Aβ-related gliosis in the mouse brains, emphasizing the significant impact of ApoE4 on Aβ during the seeding stage that may occur by perturbing Aβ clearance and enhancing Aβ aggregation [68].

The major ApoE receptors are low-density lipoproteins (LDL) receptors (LDLRs), LDL receptor-related protein 1 (LRP1), and heparan sulfate proteoglycan (HSPG), and they mediate cellular uptake of Aβ and ApoE [69]. LDLR overexpression considerably decreases ApoE levels, demonstrating its role in ApoE catabolism [69–71]. Preliminary results indicate that overexpression of LDLR LRP1 mediates cellular Aβ uptake in neurons, astrocytes, and microglia [70, 72]. In addition, LRP1 deficiency exacerbated amyloid pathology in amyloid mouse models by suppressing cellular Aβ uptake and lysosomal degradation [73]. Finally, ApoE4 is assumed to exacerbate Aβ pathophysiology by mechanisms depending on neuronal LRP [74].

### Potential protective role of APOE ε3 and APOE ε2

To better understand the potential protective role of *APOE ε3* and *APOE ε2*, clinical observation of patient with a *PSEN1 E280A* variant provides insight. This rare variant was initially identified in the largest ADAD kindred to date [75]. This amino acid substitution is known to cause Aβ overproduction and subsequent early neurodegeneration, cognitive decline, and eventually dementia. Recently, a female carrier of this variant was identified who did not develop MCI until her seventies, i.e., three decades after the expected age of clinical disease onset [75–78]. Remarkably, a [^11^C]-PiB-PET scan revealed an unusually pronounced accumulation of cerebral amyloid plaques, much higher than that detected in other cognitively impaired young mutation carriers [79]. Whole-exome sequencing demonstrated that this carrier had two copies of *APOE* containing the rare Christchurch mutation *R136S*, a variant with known a protective effect likely due to a loss of normal ApoE function [79, 80]. This *APOE ε3ch* homozygosity was assumed to delay ADAD onset whereby the protective allele’s homozygosity promotes significant resilience to highly penetrant ADAD clinical onset, possibly mediated by mechanisms limiting tau spreading and pathology even in the presence of substantial accumulation of amyloid plaques. This effect may be associated with an altered affinity for HSPGs [79]. Therefore, the degree of affinity of ApoE for HSPGs might be a factor in triggering downstream neurodegeneration.

The *APOE ε2* allele is associated with a lower risk of AD-related neurodegeneration [81, 82]. *APOE ε2* carriers show a lower risk and delayed age of onset of AD compared with *APOE ε3* homozygotes and *APOE ε4* carriers [83]. Besides reduced AD-related pathological burden, greater cortical thickness and less age-related cognitive decline are associated with the protective effects of the *APOE ε2* allele [81]. APOE ε2 was defined as an AD age-of-onset ‘decelerator’ since its variant *rs7412* delayed age-of-onset by around 12 years [84].

### Epigenetic, transcriptional, and post-translational alteration of APP and related genes

Epigenetic dysregulation—including histone modifications, DNA methylation, chromatin remodeling, and non-coding RNAs—is assumed to underlie aging-related functional decline which is itself a risk factor for several sporadic diseases, including cancer and AD [85, 86]. Human neuropathological and omics-based studies show that (i) *APP* mRNA is highly expressed in neurons, (ii) patterns of *APP* expression and the mechanisms of regulatory transcription change throughout the lifespan with an age gradient toward dysfunction, and (iii) *APP* expression is upregulated in AD brains [87–89]. DNA methylation changes in the AD brain are observed where DNA methylation of *APP* gene promoters differs from one brain region to another, with CpG island hypomethylation of the *APP* gene in AD brain tissue [90]. Differential DNA methylation is reported in other Aβ-related genes too. For example, the *DSCAML1* enhancer region was recently shown to be hypomethylated in AD brain, which in turn, was correlated with the upregulated expression of nearby *BACE1* genes [91]. In addition, histone acetylome changes in AD brain include differential H3K27-Ac peaks near *MAPT* encoding tau protein and hypoacetyl peaks downstream of *APP* and *PSEN1/2* [92].

MicroRNAs (miRNAs) constitute a large family of small non-coding RNAs that exert an inhibitory effect on gene expression by destabilizing messenger RNAs and inhibiting the translation process [93]. Mouse models and human postmortem studies indicate that the deregulation of miRNA turnover has been linked to impairment of the Aβ pathway by either upregulation of the *APP* gene or increased activity of BACE1; for other miRNAs generally related to Aβ and AD, in mice and humans, a more detailed discussion can be found in other review articles [94].

As a transmembrane protein, APP is a glycosylated protein with constitutive cell surface insertion [95]. While phosphorylation of the C-terminal fragment of APP was previously shown to alter γ-secretase processing in vitro [96] and glycation of Aβ is important for aggregation (discussed in the following sections), it is not known what other post-translational modifications of APP influence its proteolytic processing in AD brain.

### Transcriptomic response to Aβ in neuronal and glial cells

Technological advances in single-cell and single-nucleus RNA sequencing have significantly added to understanding cell type-specific changes in AD at cell level resolution in both neuronal and non-neuronal cells [97–100]. Recent data from single-cell analyses in AD mouse models and post-mortem brain from AD patients have highlighted the involvement and contribution of glial cells in AD and have led to the identification of glial subtypes that are associated with the disease, such as AD-associated microglia [101, 102] or astrocyte [103] subpopulations. The resolution offered by single-cell technologies provides an unprecedented opportunity to examine the molecular pathways and cellular processes that are associated with Aβ pathophysiology in a cell-type specific manner—particularly systematic cellular changes to the inflammatory response in microglia and astrocytes that reflect complex neuroimmune interactions in AD pathophysiology and novel disease risk genes [104].

## APP Processing: Amyloidogenic and Non-amyloidogenic Pathways

### APP cleavage and Aβ generation

**Fig. 3:**
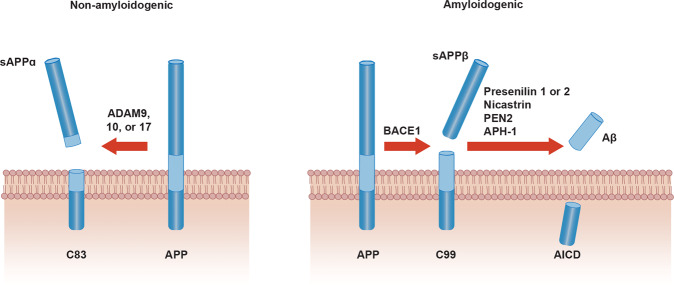
Amyloidogenic vs non-amyloidogenic pathway. Amyloid Precursor Protein (APP) is a single transmembrane protein. For the non-amyloidogenic pathway (left), APP is cleaved by A Disintegrin And Metalloprotease (ADAM) family proteases to yield the membrane-tethered C83 fragment and extracellularly released soluble APP alpha (sAPPα). In the amyloidogenic pathway (right), APP is first cleaved by β-secretase (β-APP-cleaving enzyme-1 or BACE1). CTF-β fragment is subsequently cleaved by γ-secretase composed of Presenilin 1 or 2, Nicastrin, PEN2 and APH-1. This proteolytic processing releases amyloid-β into the extracellular space. APP intracellular domain (AICD) from the initial β-secretase cleavage is released into intracellular space. Adapted with permission from ref. [392].

Three main proteases—α-, β- and γ-secretases—are involved in APP processing through (1) the amyloidogenic pathway promoting Aβ production through sequential cleavage by β- and γ-secretases, and (2) the non-amyloidogenic pathway in which APP is cleaved in the middle, either generating soluble APPα directly by α-secretase or generating shorter Aβ species such as Aβ1-15 and Aβ1-16 by the sequential cleavage by β-secretase and α-secretase. The two pathways lead to different by-products with different intrinsic functional properties, putative physiological roles, and pathophysiological implications (Fig. 3) [15, 17, 18]. Besides secretase activity, APP trafficking due to the secretory pathway is another essential factor in APP metabolism. APP is first matured in the endoplasmic reticulum and the Golgi apparatus, then translocated to the cell surface. Alternatively, APP can enter the lysosomal pathway and undergo proteolytic degradation [105].

BACE1 is the β-secretase enzyme that cleaves the extracellular juxtamembrane region of APP (β-cleavage). Cleavage of APP by β-secretase liberates the soluble N-terminus of APP (sAPPβ) while the C-terminal fragment (CTF-β or C99) remains bound to the membrane. Two mutations at the β-secretase cleavage site of APP (the Swedish mutation KM/NL and an Italian variant A673V) are linked to EOAD, and are mechanistically linked to higher sAPPβ levels due to a putatively stronger affinity of BACE1 for the changed recognition motif in APP [32, 106]. Conversely, the APP variant A673T has been reported to protect against AD due to the lower affinity of BACE1 for the APP binding site [107]. High BACE1 enzymatic activity is found in human AD brain extracts, consistent with experimental evidence of neurons producing higher levels of Aβ in AD than ‘normal’ aging [108]. BACE1 is also accumulated in dystrophic neurites close to Aβ plaques, both in AD amyloidogenic mouse models and AD brains [109–111]. Inducing autophagy in human mutant neurons promotes retention of BACE1 in distal axons, leading to the enhanced β-cleavage of APP [112].

To produce Aβ, the CTF-β fragment produced by β-secretase cleavage of APP is subsequently cleaved by β-secretase, which then releases Aβ into the extracellular space and the AICD into the cytoplasm [108]. γ-secretase is an aspartyl-type protease membrane protein complex and consists of different several components. The catalytic elements of the membrane-embedded tetrameric γ-secretase complex are represented by presenilins 1 and 2, and intramembrane-cleaving proteases responsible for generating the Aβ carboxyl terminus from APP [113, 114]. Three other proteins accounting of the complex are (i) Nct and (ii) Aph1, thought to underlie formation of a stable, high-molecular-mass protein complex supporting the catalytic activity [115, 116], and (iii) Pen-2, hypothesized to regulate the endoproteolysis of presenilins to form a stable heterodimer that binds to the Nct/Aph1 complex [108]. Besides their function in the γ-secretase proteolytic activity, presenilins participate in fundamental cellular pathways, including cell differentiation, intracellular signaling (including anti-apoptosis) [117], and membrane trafficking [105, 118].

Presenilins play a critical role in maintaining cellular homeostasis and function by modulating membrane protein degradation, intracellular vesicle/protein trafficking, lysosomal activity, and autophagy [105, 110]. More than 90 type-I transmembrane proteins have been identified as substrates of the γ-secretase complex, with the most prominent substrate aside from APP being the NOTCH receptor. Processing of NOTCH by γ-secretase liberates the NOTCH intracellular domain, which translocates into the nucleus and regulates transcription of target genes involved in cell fate decisions during embryogenesis as well as adulthood. Abrogation of NOTCH receptor processing and signaling causes dramatic phenotypes in a variety of organisms [105, 110].

In a parallel competing non-amyloidogenic pathway, APP is cleaved either by α-secretase or η-secretase to release two additional variants of the APP ectodomain, namely sAPP-α and sAPP-η [119]. Juxtamembrane cleavage of APP by α-secretase precludes Aβ generation. In vitro studies have shown that several members of the ADAM (a disintegrin and metalloprotease) family of proteases—including isoforms 9, 10, and 17—display α-secretase activity [120]. In addition, recent evidence indicates that ADAM10 is the major α-secretase responsible for the ectodomain shedding of APP in the mouse brain and likely to be active in humans [112, 121]. The η-secretase pathway is an alternative rescue pathway when BACE1 is inhibited, causing a functional shift with increased Aη-α activity and subsequent lowering of neuronal activity by an unknown mechanism [113].

### Physiological roles of APP

The expression of APP as a type I transmembrane protein is high in neurons, especially at the synaptic level. Although a full understanding of its biological function remains elusive, experimental evidence indicates a potential role in dendritic spines remodeling, molecular pathways of neurotransmission, and synaptic homeostasis [111, 122, 123]. Rescue experiments in APP KO mice show that sAPPα is sufficient to restore defects in spine density, long-term potentiation, and spatial learning [124, 125]. Most of the ectodomain shedding of APP is performed by α-secretase, which, as mentioned above, cleaves APP in the Aβ sequence, generating peptides mostly without aggregation or toxicity [126].

Although in vitro evidence suggests that soluble sAPPα has a higher impact on neural plasticity than sAPPβ [127], both peptides modulate basal synaptic transmission and short-term synaptic facilitation through binding to the GABAB receptor subunit 1a (GABABR1a) at the synapse [122]. The sushi domain of the GABABR1a binds to the full-length APP intracellularly [122], likely triggering a crucial mechanism for axonal trafficking of the complex and regulation of receptor exhibition at the presynaptic terminals. Delivery of the complex to the axonal cell surface diminishes the pool of APP available for BACE1 processing in endosomes and lowers Aβ production [122].

Aβ is an ancient neuropepetide, highly conserved across vertebrate taxa over at least 400 million years. The human Aβ sequence is shared by 60–70% of vertebrates [128], underscoring that this peptide has critical physiological functions. Aβ monomers, which are generated from the proteolytic processing of APP, can trigger or sustain intracellular signaling essential for neurotransmission, including the regulation of the excitation/inhibition balance, and synaptic vesicle trafficking [129–131]. In addition, Aβ monomers can initiate pathways mediated by the cyclic adenosine monophosphate response element-binding protein (CREB)-mediated transcription of the brain-derived neurotrophic factor (BDNF) axis, known to be involved in hippocampal neurogenesis, a key process for adult synaptic plasticity (i.e. a set of activity-dependent and adaptive structural/functional changes in synaptic strength or efficacy) [132, 133]. Loss of BDNF activation and decline of hippocampal neurogenesis have been observed in human AD dementia and MCI-AD patients, suggesting that hippocampal neurogenesis may be an early event in the synaptic failure characterizing AD [133]. Aβ released at the synaptic cleft has a critical role in sustaining neuronal bioenergetic levels essential for proper synaptic activity [134]. Experimental models of aging and AD indicate that Aβ-mediated molecular pathways are linked to lipid homeostasis and angiogenesis [135].

## Aβ Clearance Mechanisms: a Focus on the Role of the Blood–Brain Barrier

The average fractional rates of Aβ production and clearance in cognitively healthy adults are estimated to be around 8% per hour, as assessed using stable isotope labelling kinetics (SILK) technology and measurements in the CSF [136]. It is hypothesized that small reductions in Aβ clearance from the brain are sufficient to cause Aβ accumulation since efficient clearance is vital for Aβ homeostasis and preventing its toxic accumulation in misfolded assemblies given continual APP processing and Aβ generation [136]. As with all other brain metabolites, the normal average Aβ turnover depends, in part, on bulk-flow via the CSF across the blood–brain barrier (BBB), the perivascular circulation, and the glia-lymphatic (glymphatic) system in the brain [136, 137]. Moreover, multiple molecular pathways and cellular machinery are involved in the clearance process beyond the CNS, with the BBB being of crucial importance in Aβ homeostasis and clearance dynamics. In physiological conditions, the BBB protects the CNS from exposure to toxic metabolites in the systemic circulation and maintains the highly regulated brain internal milieu. Conversely, BBB anatomical disruption and functional breakdown may be detrimental for Aβ homeostasis as a part of early pathophysiological alterations in AD individuals [138].

### Aβ clearance through endothelial cells and pericytes

The core structure of the BBB is represented by endothelial cells connected by tight junctions, astrocytic end-feet, pericytes, and smooth muscle cells that ensure a selectively permeable system [139]. Soluble Aβ is transported across brain endothelial cells and transferred to the systemic blood stream mainly via LRP-1 [140] and ABC transporter sub-family A and B member 1 (ABCA1 and ABCB1 respectively) where ABCB1 on the abluminal side of the brain endothelium directly clears Aβ into systematic circulation in an ApoE-dependent fashion [139, 141].

Free Aβ can be transported from the circulation into the interstitium via receptors for advanced glycosylation end-products (RAGE). Soluble transporters (known as ‘sequestering agents’; including soluble forms of RAGE (sRAGE) and LRP (sLRP)) bind to soluble Aβ and inhibit its binding to RAGE, thereby preventing Aβ from entering the interstitium [139, 141]. Preliminary results indicate that, in AD, expression of the blood efflux transporters LRP1 and ABCB1 is decreased, whereas expression of the blood influx transporter RAGE is upregulated [139, 141].

### Aβ clearance through intracellular and extracellular enzymatic degradation

There is preliminary evidence showing that intracellular Aβ can be degraded by proteasomes and Aβ-degrading enzymes (ADE) via the ubiquitin-proteasome pathway in neurons and the extracellular neprilysin–mediated pathway, respectively [142]. Mouse models of AD indicate that components of the ADE system can be impaired [139, 142] and that Aβ can inhibit the proteasome, through cross-pathways influences, including a lysosomal cathepsin B-mediated mechanism [143]. Therefore, experimental data suggest the existence of a self-reinforcing detrimental protein homeostasis cycle [143, 144].

The ADE encompasses the zinc metalloendopeptidase (NEP-1 and NEP-2, endothelin-converting enzyme (ECE)-1 and -2, angiotensin-converting enzyme (ACE)), thiol-dependent metalloendopeptdiase (insulin-degrading enzyme (IDE)), serine proteases (plasmin, myelin basic protein and acylpeptide hydrolase), cystein proteases (cathepsin B, D, and S), matrix metalloproteinase (MMP-9, MMP-2), Kallikrein-Related Peptidase 7 and others (GCPII, aminopeptidase A, mitochondrial peptidasome) [145, 146–148]. Many genes identified through GWASes and established as risk factors for AD are linked to Aβ degradation through the endosomal-lysosomal system (RIN3) or ubiquitin-proteasome pathway (CLU and PTK2B) [39, 40].

### Aβ clearance via brain interstitial fluid (ISF) bulk-flow and CSF absorption

The perivascular drainage pathway has a significant role in ISF bulk-flow clearance of Aβ [19]. Failure of perivascular drainage of Aβ and increased Aβ deposition in arterial walls has two detrimental downstream effects: (a) microbleeds due to rupture of Aβ-laden arteries, namely cerebral amyloid angiopathy that has high comorbidity with AD, and (b) AD itself where the failure of elimination of ISF, Aβ, and other soluble metabolites from the brain alters homeostasis and the neuronal micro-environment, and is associated with synaptic decline and cognitive-functional impairment.

The glymphatic system was proposed as a CSF-ISF exchange system in absence of direct lymphatic access to the brain and with astrocytes as cellular links between brain parenchyma and the perivascular pathway, with eventual solute transport to the cerebrovenous network and meningeal lymphatic vessels [149]. While there is limited knowledge of the anatomy and function of the glymphatic system in humans, mouse models of aging and AD show that the glymphatic pathways represent a vital clearance system for driving the removal of soluble Aβ from the interstitium [149]. Several other glymphatic-related factors with implications for AD include expression and localization of aquaporin 4 (AQP4) channels on astrocytic endfeet, arterial pulsation, and diurnal glymphatic cycles corresponding to sleep-awake rhythms [150–152].

CSF absorption clearance of Aβ occurs via both circulatory and lymphatic systems. Such processes depends on CSF production by the choroid plexus, blood-CSF barrier structural integrity, relevant transporters, arachnoid villi resistance, and CSF lymphatic absorption, all of which decline with age [153]. In AD, the blood-CSF barrier structural integrity is affected and associated with aberrant Aβ clearance [154]. Both increased CSF outflow resistance at the arachnoid villi level and decreased lymphatic CSF absorption have been reported as brain aging alterations and primary risk factors for AD.

## Aβ Biochemical Properties from Monomers through Higher Aggregation States, Including Plaques

**Fig. 4:**
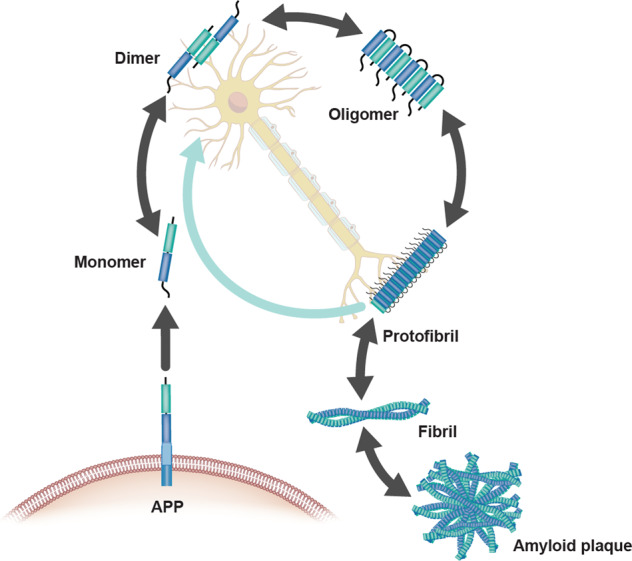
Amyloid-β aggregation species and evidence of reversible states: the amyloid-β cycle. Aggregation species of Aβ can exist as monomers, dimers, oligomers, protofibril, fibril and amyloid plaques. These species exist in steady state where one form can convert to another in a bidirectional manner. The species are characterized by aggregate size, conformation state and solubility, with fibril and amyloid plaque being insoluble. Adapted with permission from ref. [108].

After being generated as soluble monomers, Aβ is found in several different intermediate aggregation states, including dimers and trimers, soluble oligomers, and protofibrils, until it forms fibrils that accumulate in plaques, typically viewed as an AD neuropathological hallmark (Fig. 4). Understanding the biology and interlinked dynamics of these intermediate assemblies and their bio-activity, in either physiological and pathophysiological conditions, is essential from a diagnostic and therapeutic perspective.

### Monomers

As reported above, in physiological conditions, Aβ monomers are involved in neuronal cytoprotective pathways as well as intracellular signaling and synaptic functions [122, 123]. The molecular dynamics underlying the incipient Aβ monomer self-assembly are not known though some in vitro and animal models have provided plausible preliminary hypotheses.

Albeit observed only in vitro, the aggregation of Aβ involves a series of interconnected processes, which starts with a primary nucleation step leading to the formation of disordered oligomers that then convert into growth-competent nuclei [155]. These nuclei can then elongate into fibrillar assemblies, which catalyze the formation of new nuclei, in a feedback process known as secondary nucleation, responsible for the proliferation of the aggregates [156].

Aβ1-42 is less soluble than Aβ1-40 and thus more likely to form aggregates. In this regard, protein solubility has emerged as a critical aspect of protein homeostasis as proteins generally evolved to maintain the solubility required for their optimal function [157, 158]. A variety of aspects of Aβ homeostasis can affect Aβ aggregation. For example, glycation appears as a relevant early event that stimulats amyloid aggregation, followed by increased protease resistance and insolubility [159]. Proteins in amyloid deposits, like Aβ, are frequently glycated [160], suggesting a direct correlation between protein glycation and amyloidosis as well as a link to diabetes [161]. Advanced glycation end-products (AGEs)-modified Aβ peptide-nucleation can seed accelerated aggregation of soluble Aβ peptide versus non-modified seed material [162]. N-terminal truncations of Aβ are less soluble, more prone to aggregation and associated with enhanced toxicity [163], in particular pyroglutamylated variants form when N-terminal truncations expose a glutamate residue which is then transformed into pyroglutamate by the enzyme glutaminyl cyclase [164]. By contrast, the oxidation of methionine 35 increases the solubility of the C-terminal region of Aβ and reduces the aggregation propensity of the peptide [165].

### Soluble oligomers

Soluble Aβ oligomers are biochemically defined as Aβ assemblies that are not pelleted from physiological fluids by high-speed centrifugation [166]. Generally, soluble protein misfolded oligomers of unrelated sequences share characteristic structural features with specific immunoreactivity, distinct from those of monomers and fibrils [167]. Soluble Aβ oligomers derived from human brains have molecular weight distributions corresponding to a mixture of dimers to dodecamers [168, 169]. Intracellular and secreted soluble dimeric and trimeric Aβ oligomers were observed in human-derived neurons, as well as APP transgenic mouse models [156, 170, 171]. Mass spectrometry studies have shown that brain-derived bioactive 7 kDa Aβ species are composed of a heterogeneous mixture of covalently cross-linked dimers of different Aβ fragments, which might represent the smallest synaptotoxic species [172, 173].

Robust evidence for the toxic potential of soluble Aβ oligomers derives from studies showing that soluble, low-number oligomers of naturally secreted human Aβ injected in rodent hippocampus can hinder the activity-dependent modulation of synaptic strength and long term depression (LTD) (i.e., synaptic plasticity) [172, 173]. In particular, different Aβ species—including soluble, low-number oligomers—can inhibit key electrophysiological and ultrastructural mechanisms of synaptic plasticity, such as long-term hippocampal potentiation (LTP), enhance LTD and lead to synaptic loss as assessed by the decrease of dendritic spine density [174]. With cell-derived Aβ oligomers, this inhibition occurs at low- to sub-nanomolar concentrations similar to those found in human CSF [175, 176]. Experimental models of AD showed that low-number Aβ oligomers obtained intracellularly from APP-expressing cultured cell lines, disrupt hippocampal LTP in brain slices and in vivo, impair memory of complex learned behavior in rats, and decrease dendritic spine density in organotypic hippocampal slice cultures [177]. Larger aggregates such as dodecamers also exhibit substantial neurotoxicity [178].

Aβ oligomers promote a rapid decrease in membrane expression of memory-related receptors, followed by abnormal spine morphology, reduction in spine density, and synaptic deterioration in cultures of hippocampal neurons [179]. Experiments performed with brain-derived oligomeric species provided a highly diversified picture, supporting the existence of a mixture of water-soluble Aβ species promoting synaptotoxicity [180]. Experimental data in AD extracts show that low molecular weight Aβ oligomers, which are the most aqueously diffusible, effectively mediated disruption of both neuronal structure (neurite integrity) and function (synaptic plasticity), suggesting that only a small pool of toxic Aβ oligomers displays bioactivity [180, 181].

### Protofibrils

During the aggregation of monomeric Aβ to insoluble fibrils, several intermediate species are formed, including large soluble aggregates known as protofibrils, as described by Walsh and colleagues [182]. These protofibrils were defined as the soluble oligomeric species of synthetic Aβ peptides appearing as a peak in the void volume (>75 kDa) of a size exclusion chromatography with a Superdex G75 column [183, 184]. Such soluble Aβ species have been shown to induce electrophysiological changes, and neurotoxicity in rat cortical neurons [185]. Aβ protofibrils inhibit LTP-mediated synaptic plasticity in mouse hippocampus, thus impairing pivotal cognitive/behavioral functions such as spatial-temporal pattern separation and learning processes [186]. Aβ protofibrils can accumulate in glial cells, are associated with inflammatory responses, and are present in activated astrocytes in AD brains [187]. In cultured microglia in vitro, Aβ protofibrils are internalized by microglia more extensively than monomers [188]. They can further be released through microglia-derived microvesicles, possibly contributing to extracellular spread and neuroinflammation [189]. A peripheral immune response to the toxic Aβ protofibrils is suggested by the observation that the number of B cells producing auto-antibodies against Aβ protofibrils is significantly higher in AD patients than healthy controls [190].

Soluble protofibrils of various sizes have been identified in human brains and in brains from APP transgenic mice [191–193]. However, it is still unclear which particular aggregated soluble Aβ species confer toxicity. The detrimental agents may consist of high molecular weight and low molecular weight soluble Aβ aggregates with distinctive conformations.

An important model for the study of protofibrils is the Arctic *APP* mutant (*APP E693G*) which causes EOAD, and has been shown to specifically increase the rate of formation of these species [183, 184, 194]. In ArcSwe transgenic mice, a model with both the Swedish and the Arctic mutations and expressing abundant levels of protofibrils, cognitive deficits were shown to occur without plaques accumulation and concomitantly with the detection of early and widespread punctate (grain-like) intraneuronal Aβ-immunoreactive staining, as indicated by highly selective N-terminus 6E10 [epitope 1–16] and 3D6 [epitope 1–5] Aβ-antibodies. Such intraneuronal peptides are hypothesized to reflect intracellular non-fibrillar Aβ aggregates (protofibrils, given the underlying Artic mutation). Intraneuronal peptides predated parenchymal plaques accumulation [195]. Levels of Aβ protofibrils in the brain, but not of total Aβ, correlated with spatial learning, adding further evidence to the hypothesis of soluble protofibrils being the most toxic Aβ species [196]. The pool of soluble toxic Aβ assemblies consists of particles in the size range of 75–500 kDa [197]. Such species are selectively detected by the murine equivalent of BAN2401, mAb158, a protofibril-targeting antibody with low binding to monomers and insoluble Aβ fibrils [193, 198]. Importantly, mAb158 has been shown to significantly reduce protofibril levels in the brain and CSF from ArcSwe transgenic mice after chronic treatment [199].

Studies of AD patients with the Arctic mutation showed that they were, as expected, negative for fibrillar Aβ, as measured by the brain retention of the amyloid ligand Pittsburgh compound B ([^11^C]-PIB) with positron emission tomography (PET) [200]. A novel pathogenic *APP* mutation (*E693del [Osaka]*) was identified in Japanese pedigrees with AD, producing an Aβ variant—E22Δ—lacking Glu22 [201]. The E22Δ peptide variant was more resistant to proteolytic degradation and had the distinctive aggregation property of enhanced oligomerization (but no fibrillization) [201]. In vivo studies in rats demonstrated more effective hippocampal LTP inhibition by E22Δ peptide versus the wild-type Aβ peptides [201].

Taken together, and based on the current knowledge of underlying disease mechanisms, various soluble Aβ aggregates, and specifically, Aβ protofibrils, are particularly harmful and should be a compelling therapeutic target in AD.

### Fibrils and plaques

Under physiological conditions, amyloidogenic proteins and peptides—such as Aβ—spontaneously aggregate into amyloid structures in a concentration-dependent manner. This phenomenon is general since, at the concentrations typically found in the cellular environment, proteins are metastable only in their native states [157, 158]. The conversion into the more stable amyloid state is prevented by the presence of high free energy barriers [157, 158]. In AD, specific brain micro-environmental conditions—including a vulnerable protein homeostasis system [202] and the abundance of a variety of poorly soluble proteins—appear to facilitate the formation of Aβ fibrils. Aβ fibrils form the characteristic cross-β-sheet structure of amyloid fibrils, in which Aβ peptides assemble into β-sheets with β-strands perpendicularly oriented to the long axis of the fibril and stabilized by hydrogen bonds [203–207].

Aβ fibrils are polymorphic with molecular structures that depend on the aggregation conditions [208]. Structurally distinct fibrils can have different levels of solubility, accumulation rates, and toxicity levels in neuronal cell cultures [206, 208]. Aβ fibrils, and to a lesser extent plaque, are associated with synaptic dysfunction in AD animal models and in AD patients. Fibrillar Aβ deposits are observed in the vicinity of disrupted neurites [209], of regions of decreased spine density, and in areas of neuronal loss [206, 210]. Moreover, primate models of AD show that microinjection of Aβ fibrillar assemblies in the cerebral cortex causes neurodegeneration, neurofibrillary pathology, and neuroinflammation [211]. These observations are consistent with the finding that Aβ fibril surfaces can catalyze the formation of Aβ oligomers [156], and Aβ oligomers have been observed surrounding Aβ fibrils [212].

### Rates of recycling of the Aβ aggregation states

The interconversion of Aβ monomers, oligomers, protofibrils, and amyloid fibrils is implicated in AD pathogenesis [213]. By inspecting the nature of the amyloid fibrils structure, a continuous process of dissociation and re-association, resulting in the recycling of molecules within the fibril pool was observed [214]. Determining the kinetics of the individual association and dissociation reactions are challenging since the forward and reverse reactions to and from different Aβ aggregation states co-occur [155, 157, 213, 215]. Likewise, the heterogeneous set of oligomers consists mainly of unstable aggregations that can dissociate back to monomers but includes assembling species as well. Oligomers undergo repeated cycles of formation–dissociation before eventually turning into species that can grow into new fibrils [155].

Molecules making up Aβ1-40 fibrils recycle to a much greater extent than those of Aβ1-42. The rate constant for dissociation of molecules from the fibril is much higher for Aβ1-40 compared with Aβ1-42 [215]. Typically, the N-terminal region of Aβ contributes to improving fibrillar stability due to a gain of function mechanism at low pH, specifically at the pH range found within the endosomal and lysosomal pathways [216]. Along with pH, brain lipids play a critical function in destabilizing and rapidly re-solubilize mature Aβ fibers. This equilibrium is not reversed toward monomeric Aβ but, instead, toward soluble Aβ protofibrils [217]. A balance has been found between relatively inactive intermediate-sized Aβ aggregates and highly cytotoxic Aβ aggregates such as small oligomers and large protofibrils, which may have an impact on the role of amyloid plaques in the pathogenesis of cellular dysfunction in AD [181].

## The Toxicity of the Aβ Pathway

Biomarker-based studies conducted in EOAD and LOAD have shown a temporal sequence between incipient Aβ pathophysiology, spreading of Aβ aggregation species and plaques through brain areas, and eventually increase of tau and neurodegeneration-based biological signatures [6, 8, 17, 54]. Although no causal effect has been established between Aβ pathophysiology and AD-related pathophysiological changes taking place at different temporal scales, a body of experimental and in-human studies indicates that Aβ aggregation species may exert a permissive/facilitating role on other pathophysiological pathways and/or unfold synergistically with them [8, 17, 76].

### Aβ pathophysiology and tauopathy

**Fig. 5:**
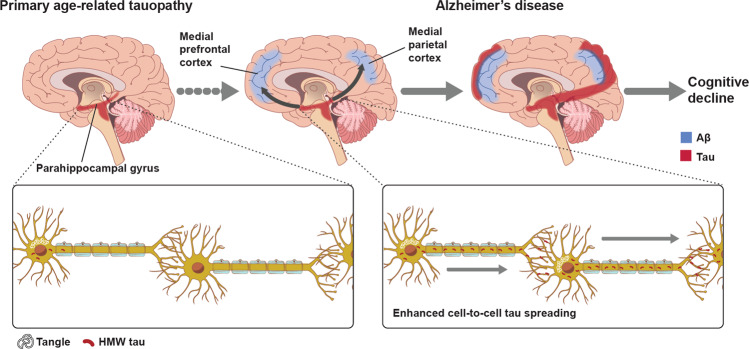
The evidence-driven experimental model of Aβ-tau synergy. Accumulation of neurofibrillary tangles made up of tau (red) and amyloid plaques composed of amyloid-β (blue) coincides in the neocortical areas in the brain of Alzheimer’s disease subjects supporting amyloid-β dependent tau propagation across neocortical regions. Inter-neuronal spreading of tau (bottom) is enhanced in AD brains with both plaques and tangle build-up. Adapted with permission from ref. [221].

The spatial-temporal relationship between the Aβ pathway and tau pathophysiology in AD, at both the molecular and macroscale, is critical to understanding AD pathogenesis and pathophysiological progression, and has gained momentum recently with the validation of several biomarkers charting different biological levels. The currently most accepted model indicates that Aβ pathophysiology may be an upstream pathophysiological event in AD and may function as a trigger/facilitator of downstream molecular pathways, including tau misfolding, tau-mediated toxicity, accumulation in tangles, and tau spreading that leads to cortical neurodegeneration (see Fig. 5) [218–222]. Genetic studies support biomarker-based observations and experimental studies which indicate a temporal Aβ–tau synergy where there is a pathophysiological sequence between aggregation of Aβ and tau-mediated toxicity [221].

In a study of the Colombian ADAD kindred with PSEN1 E280A mutation carriers who were age- and sex-matched to mutation non-carriers, the onset of cortical Aβ deposition was around 15 years before dementia onset [223]. Notably, one mutation carrier exhibited tau-PET pathology in the medial temporal entorhinal-cortical area around 6 years before the estimated clinical symptoms onset, suggesting a 10-year gap between the development of Aβ pathology and tau-PET pathology [76]. Tau-PET pathology was not present in ADAD mutation carriers if Aβ cortical levels did not exceed the clinical disease threshold. Evidence suggests that the highest tau amounts detected by PET were found in those with the highest amyloid plaque pathology [76].

In sporadic AD, neuroimaging studies show that cortical tau-PET ligand retention is increased only in the presence of cortical Aβ accumulation and is associated with cortical thinning in AD [224]. Longitudinal studies show that a fast rate of antecedent Aβ accumulation predicts subsequent tau deposition in the inferior temporal cortex [225]. In the last 10 years, extensive research effort has been dedicated to understanding whether Aβ represents a trigger or a driver of AD, or both. Most of the studies report that tau markers, more than Aβ markers, significantly covary with neurodegeneration markers and long-term cognitive/functional outcome measures suggesting that Aβ pathophysiology triggers downstream pathways including tau-mediated toxicity and facilitates tau spreading [17, 214, 215]. These results, supported by experimental evidence (see below) suggest that AD is an Aβ-facilitated tauopathy leading to cognitive decline, MCI, and dementia. According to these PET-based investigations in both ADAD and LOAD, Aβ pathophysiology is likely to play a role in fostering the development of tau pathology.

Experimental models indicate that soluble forms of Aβ and tau synergize to exert synaptic toxicity independently of their assembly into plaques and tangles. Mouse models of AD show that modulation of tau seeding is associated with lower neurodegeneration rates and memory deficits without significant changes in the level of brain Aβ accumulation [226]. The triple transgenic mouse model (3xTg-AD) displays increasing extracellular Aβ accumulation in the neocortex and hippocampus before the seeding of tau into tangles [227].

Crossing familial AD-mutant APP mice with mutant MAPT transgenic mice leads to enhanced tau pathology and supports the occurrence of tangle-like alterations downstream of Aβ accumulation [220]. Injection of Aβ fibrils into P301L mutant tau transgenic mice’s brains triggers a five-fold rise in NFTs in cell bodies within the amygdala from where neurons project to the injection sites [222]. Crossing transgenic mice showing the spread of tau from the entorhinal cortex to other brain regions with APP/PS1 mice revealed that cortical amyloid deposition caused a dramatic increase in tau spreading to distal brain regions [228]. These findings support the hypothesis that cortical Aβ is permissive for the spread of tangles from the medial temporal lobe associated with cognitive decline in AD. According to the Braak neuropathological staging, such a pathophysiological model fits in the amyloid-independent progression of tau pathology [220].

Several findings deriving from mouse models converge toward an upstream role of Aβ on tau dyshomeostasis by facilitating/promoting tau conversion from a normal to a toxic state that can enhance Aβ toxicity via a feedback loop [228, 229]. Critical insights derive from in vitro studies. Tau hyperphosphorylation is promoted by synthetic Aβ oligomers and soluble extracts containing Aβ oligomers from AD brains (but not in non-AD brains) [230]. Treating healthy rat neurons in culture with soluble Aβ oligomers isolated from the AD cortex generated neuritic dystrophy and AD-type tau hyperphosphorylation. However, no dystrophy followed if tau expression was first knocked down [231]. Other similar studies suggested that Aβ, particularly soluble oligomers of Aβ1-42 [222], could trigger AD-type tau alterations, supporting the sequence that human genetics indicated. EOAD mutations in APP and PSEN1 promotes Aβ extracellular deposition, including Aβ plaques, in a human neural stem-cell-derived-3D culture system [232]. Cells expressing familial AD mutations exhibited high hyperphosphorylated tau levels in both the soma and neurites. In summary, there is extensive experimental evidence implying that inhibition of Aβ generation would be expected to decrease Aβ pathology and attenuates tauopathy [221].

### Aβ pathophysiology and neuroinflammation

The spatiotemporal relationship between Aβ and glial cells, which are the critical orchestrators of neuroinflammation, is a rapidly expanding area of research to determine whether neuroinflammation can trigger and sustain incipient Aβ dyshomeostasis, or compensate for it, or carry out both in a stage-dependent manner. To date, most of the studies in vitro and in murine models of aging and AD support the notion that neuroinflammation is a key pathogenic event in AD etiology. The in-human exploration of neuroinflammatory mechanisms is still limited because of the early stage of development or the lack of clinical validation of relevant biomarkers.

Aβ species can interact with microglial and astrocytic pattern recognition receptors that initiate innate immunity where sustained microenvironment alterations—such as brain accumulation of Aβ—can trigger microglia “priming” [233]. Priming makes microglia susceptible to secondary inflammation stimulating factors, which can then amplify inflammatory reactions [233]. Two main phenotypical categories of microglia cells are present in the brain; resting (or quiescent) and activated. Activated microglia are typical pathophysiological features of AD and other neurodegenerative diseases [234–236].

Experimental AD models demonstrate that microglia surround plaques and fibrils, likely creating a physical barrier that can prevent their spreading and toxicity [237]. Microglia may contribute to Aβ clearance as well as limiting plaque growth and accumulation [238, 239]. Moreover, the dysregulation of microglia activity, including that from dystrophic microglia, may be a trigger and an aggravating factor of the seeding of aberrant protein aggregates in the brain [235, 236]. In AD mouse model, there is a transition from the resting to the activated states of microglia that may be the consequence of physiological stress, or Aβ triggered activation stimuli [240].

At a molecular level, inflammation is promoted by the presence of Aβ aggregates, including oligomers, protofibrils, and fibrils [241–244]. Microglia can bind to soluble Aβ oligomers, protofibrils, and insoluble Aβ fibrils through cell surface receptors, including the class A1 scavenger receptor (SCARA1), cell surface cluster of differentiation (CD) markers (CD36, CD14, CD47), α6β1 integrin, and Toll-like receptors [245–248]. Aβ species induce neuroinflammation and neurodegeneration by stimulating the microglia to release pro-inflammatory cytokines and interfering with the synthesis of anti-inflammatory cytokines such as transforming growth factor-beta1 (TGF-β1) [249–251]. TGF-β1 is a neurotrophic factor displaying both anti-inflammatory and neuroprotective actions stimulating Aβ clearance by microglia [252, 253]. TGF-β1 deficit exerts a key pro-inflammatory role in AD. A selective impairment of the TGF-β1 pathway is present in early AD, both in animal models and the human brain [242, 243, 254, 255]. Tumor necrosis factor-alpha (TNF-α) is a cytokine exerting a pivotal role in early pro-inflammatory processes in preclinical AD, as shown by both AD animal models and human longitudinal studies. In AD, TNF-α is chronically released by activated microglia, neurons, and astrocytes, and increased levels of extracellular Aβ stimulate its release [256–259]. TNF-α can stimulate γ-secretase activity, resulting in increased synthesis of Aβ peptides and a further increase in TNF-α release [249]. Animal studies highlight the association between TNF-α pathway blocking and histopathological marker reductions, such as Aβ plaques formation and microglial cell number decreases in the AD brain [260]. In humans, multiple studies detected elevated TNF-α levels in both MCI and AD dementia [260, 261].

In early AD pathogenesis, Aβ oligomers, protofibrils, and fibrils gather in the extracellular space and elicit a pathological cascade, eventually resulting in neuronal death [256–259]. Microglia eliminate these Aβ forms, as well as dying and dead cells through phagocytosis [262]. Aβ clearance can be stimulated by the release of numerous proteases participating in Aβ degradation [263]. In this regard, TREM2 modulates microglial functions by stimulating inflammatory cytokine production in response to Aβ plaques [264, 265]. The absence of TREM2 can enhance Aβ pathophysiology during early AD, which can be exacerbated by decreased phagocytic Aβ clearance in later disease stages [265], TREM2 variants reduce the Aβ phagocytic ability of microglia. TREM2 is the primary positive regulator of microglia phagocytosis, whereas CD33 is the negative regulator downstream to TREM2 [266, 267]. While additional in vivo studies will be necessary to clarify ApoE isoform-dependent function in cellular Aβ uptake and metabolism, there is evidence that microglial uptake of Aβ is facilitated by TREM2, ApoE, and CLU/ApoJ [268].

Along with microglia activation, hypertrophic reactive astrocytes can surround Aβ plaques as observed in human postmortem studies and in animal models [269, 270]. In AD, astrocytes release various pro-inflammatory molecules after exposure to Aβ (i.e., cytokines, interleukins (ILs), complement components, nitric oxide, and other cytotoxic compounds) and thus ultimately, amplify the neuroinflammatory response [260, 261, 271]. Human neuropathological studies conducted on AD brains report the presence of cytoplasmic inclusions of non-fibrillar Aβ in astrocytes, reflecting a phagocytic engulfment of extracellular Aβ deposits [260–262]. In addition, rodent models of AD indicate the astrocytes’ ability to take up and clear Aβ in individuals bearing cerebral fibrillar aggregates and diffuse plaques [260–262]. Conversely, compromise of astrocyte-mediated synaptic homeostasis is associated with increased Aβ plaque burden and synaptic terminal dystrophy [260–262]. This enhanced phagocytic activity may represent a compensatory mechanism to incipient increase in Aβ accumulation to neutralize its toxicity.

### Aβ pathophysiology and the neurochemical systems in AD: the cholinergic system

There are complex and non-linear dynamics between Aβ homeostasis and the basal forebrain’s cholinergic system, one of the earliest brain anatomical structures to degenerate in AD. Both neuropathological and neuroimaging studies conducted in cognitively healthy older adults have reported correlations between increased BACE1 activity, Aβ accumulation with basal forebrain atrophy and loss of functional connectivity [272–276], and loss of projections to other cortical sub-cortical regions [277, 278]. Such an inverse correlation is likely to be aggravated by the presence of the APOE4 allele [279]. Furthermore, those progressing from MCI-to-dementia exhibited smaller baseline basal forebrain volumes and faster basal forebrain atrophy progression versus MCI-stable individuals [280]. These findings support previous evidence on the disruption of the cholinergic basal forebrain nuclei that may precede clinical onset [281].

Complex interactions exist at the molecular level. Muscarinic acetylcholine receptor agonists (mainly M1-type; to a lesser extent M3-type) can downregulate amyloidogenic and tau-generating pathways. M1 agonists may act as functional activators of protein kinase C (PKC) signaling, which, in turn, promotes a metabolic shift towards α-secretase activity by upregulating ADAM17 (also known as TNF-α-converting enzyme or TACE) [282]. Experiments in a mouse model of AD showed that the activation of α7 nicotinic receptors leads to downregulation of glycogen synthase kinase-3 (GSK3), a kinase involved in Aβ oligomer-induced inhibition of LTP as well as tau hyperphosphorylation [283, 284]. Possibly, α7 nicotinic activity and coupling of M1 to PKC lead to a downregulation of detrimental cell processes occurring in AD, such as GSK3-mediated tau hyperphosphorylation [285].

### Aβ pathophysiology and the neurochemical systems in AD: the glutamatergic system

Glutamate excitotoxicity is considered one of the core molecular mechanisms of neurodegeneration in AD [286, 287]. The interaction between Aβ aggregates and glutamatergic neurotransmission is a possible critical event for the Aβ-induced disruption of excitatory synaptic transmission and plasticity associated with cognitive deficits [286, 287]. Aβ species can promote the dysregulation of *N*-methyl-*D*-aspartate (NMDA) and, to a lesser extent (*α*-amino-3-hydroxy-5-methyl-4-isoxazolepropionic acid) (AMPA) ionotropic glutamate receptors (NMDARs and AMPARs) in the brain [286, 287].

Electrophysiological recordings on mouse hippocampal slices showed the ability of soluble Aβ oligomers to enhance the activation of NR2B/2A subunits of NMDARs while inhibiting glutamate uptake and recycling at the synapse [286, 288]. Consequently, a partial block of NMDA receptors coupled with a shift of the activation of NMDAR-dependent signaling cascades can take place, thus inducing LTD and downstream synaptic loss. The hippocampal overstimulation of Aβ oligomers is associated with decreased cell surface expression of NMDARs (downregulation via endocytosis) and alterations of dendritic spine density [286, 288, 289].

In AD, synaptic transmission and plasticity impairment is partially due to loss of AMPARs homeostasis with unbalanced trafficking and/or turnover [290]. AMPARs are the principal receptors mediating fast excitatory synaptic transmission in the mammalian brain [291]. Dynamic trafficking of AMPARs to and from synapses is a critical mechanism underlying the induction of synaptic plasticity. Overexpression of APP and high concentrations of soluble Aβ oligomers are associated with the downregulation of GluA1/2 subunits of AMPARs and downstream impairment of synaptic plasticity, spine loss, and memory deficits [292, 293]. As with the NMDARs, the mechanisms leading to AMPARs downregulation are not fully understood [294].

### The spatial-temporal association between Aβ pathophysiology and brain networks damage

Multi-modal studies—conducted across the entire AD clinical continuum and combining molecular, structural and functional neuroimaging as well as fluid biological signatures—show a close spatial-temporal overlap between Aβ accumulation and distinct brain endophenotypes. The combination of amyloid-PET and volumetric/shape analysis MRI indicate that incipient higher rates of PET standardized update value ratios (SUVRs) are associated with hippocampal gray matter atrophy, an established biomarker of AD-type neurodegeneration, even in cognitively healthy individuals [6, 17, 295–297]. Such findings are consistent across studies investigating fluid biological signatures (CSF and or plasma Aβ species) and hippocampal volumes [298], experimental models of AD [227], human neuropathological data [6, 17, 18], and fluid biomarkers studies investigating dendritic proteins, like neurogranin, charting hippocampal disruption and synaptic dysfunction [299]. Hence, the overall evidence points toward hippocampal atrophy as a pathophysiological event observable as early as the incipient Aβ accumulation.

Selective brain structural damage—including at the hippocampal level—due to initial Aβ toxicity may occur downstream to ultrastructural changes that may underlie functional impairment [17]. In the limbic system [300, 301], the mesial temporal and superior parietal cortex [302, 303], activity change in the default-mode network (DMN) and the central executive (CEN) and the salience (SaN) networks [304, 305] is associated with worse cognitive trajectories in individuals displaying elevated Aβ burden [302, 303]. Early Aβ-associated reduction in DMN activity can take place before Aβ biomarkers (either PET or CSF) become positive, thus indicating a potential upstream toxic role of Aβ aggregation species in selectively vulnerable regions such those belonging to the DMN [306]. In prodromal stages of AD, loss of DMN functional connectivity is associated with neocortical and hippocampal gray matter volume loss, considered to reflect downstream neurodegeneration [302, 307]. As addressed above, whether this effect is necessarily tau-mediated or partially induced by Aβ species toxicity needs to be fully elucidated [308]. Eventually, lower DMN connectivity is associated with faster cortical shrinking, but only in those with elevated baseline Aβ-PET indexes [309]. This evidence in humans is supported by experimental models of aging and AD that point out the intrinsic bio-energetic vulnerability of the DMN neurons [300].

Multi-modal imaging studies show an increased Aβ-PET signal within the CEN and the SaN [300, 310, 311] throughout the biological continuum of AD and in aging. A spatial covariance between Aβ accumulation and connectivity and metabolism in the CEN and SaN (decreased) [300, 312] has been reported in AD [313–316]. The SaN plays a key role in the coordination of the DMN and the CEN, and whose functional impairment is associated with early learning and episodic memory deficits that characterize AD [317].

## Discovery, Development, Validation, and Qualification of in vivo Biomarkers

### CSF and blood-based biomarkers of Aβ: monomers

The three core AD CSF biomarkers Aβ42, total-tau (t-tau), and phosphorylated tau (p-tau) contribute diagnostically relevant information, especially during the early phases of the disease [318]. Low CSF Aβ1-42 concentrations display an average sensitivity greater than 90% for detecting cortical Aβ deposition of across all clinical stages of AD, including preclinical, prodromal, and dementia [319–322]. According to the current research diagnostic criteria, Aβ1-42 and tau (t-tau and p-tau) should be used in combination. The simultaneous presence of low Aβ1-42 and high t-tau and p-tau concentrations strongly suggests an AD diagnosis even at a prodromal stage, with a sensitivity of 90–95% and a specificity of about 90% [323]. The CSF tau/Aβ1-42 ratio represents a reliable tool for predicting cognitive decline in non-demented older adults and individuals with subjective cognitive decline, a risk factor for AD [324–326].

CSF Aβ1-42 has the potential to discriminate AD from frontotemporal lobar degeneration. Still, it shows significant overlap with other non-AD neurodegenerative diseases, specifically Lewy body disease, which is frequently characterized by concomitant Aβ pathology [299, 318]. The CSF matrix contains many different Aβ isoforms, of which Aβ1-40 is ~10 times more abundant than Aβ1-42 [327]. CSF levels of Aβ1-40 are unchanged in AD, but there is a reduction in the CSF Aβ1-42/Aβ1-40 ratio that is more marked than the decrease in Aβ1-42 alone. The CSF Aβ1-42/Aβ1-40 ratio improves diagnostic accuracy and has a better concordance with amyloid-PET positivity [299, 318, 328]. based on that CSF Aβ1-40 serves as a proxy for the ‘total’ Aβ production, thereby normalizing for differences in basal Aβ production between individuals [318, 328] or normalizing for between-individual differences in CSF dynamics or pre-analytical confounders affecting both Aβ1-42 and Aβ1-40. A marked reduction in CSF Aβ1-42 and the Aβ1-42/Aβ1-40 ratio has consistently been found in patients at different stages of AD [318, 329], and it supports the diagnostic differentiation between AD and non-AD clinical phenotypes.

From a biological standpoint, several factors—especially the APOE ε4 allele and sex—may influence the biomarker concentrations across large-scale populations [330]. However, it has been shown that the dependence of CSF Aβ1-42 levels on the APOE ε4 allele is due to carriers having more Aβ deposition; this association is not present in Aβ-negative young people [330]. From a methodological standpoint, pre-analytical and analytical factors may affect absolute levels of Aβ1-42 [331]. Unified protocols for CSF collection and handling have been published by the Alzheimer´s Association, such standardization will facilitate the introduction of globally accepted thresholds for CSF the AD biomarkers. Among the various recommendations and solutions put forward, the recent development of fully automated assays provides the basis for globally replicable and accepted cut-off points [299, 332]. Indeed, fully automated assays significantly minimize operator and lot-to-lot related variability (i.e., both intra-laboratory and inter-laboratory variability) [333].

CSF (and blood-based) biomarkers provide somewhat different information from Aβ-PET, with the latter showing the brain regional distribution of Aβ. In contrast, the former allows the simultaneous investigation of different pathophysiological mechanisms other than brain Aβ accumulation [299, 318, 331]. There is no consensus on whether CSF and PET biomarkers of Aβ accumulation become positive at the same preclinical disease stages. However, most studies show a small percentage of patients with abnormal CSF Aβ with negative Aβ-PET, and these progress to positive Aβ-PET, suggesting that CSF changes likely precede PET detection of cortical Aβ [334, 335]. A growing body of literature has demonstrated that the temporal dynamics of plasma Aβ mirror the CSF matrix [336].

### Plasma biomarkers of brain Aβ accumulation

Blood-based biomarkers are expected to facilitate critical clinical solutions catalyzed by the global threat of AD. These biomarkers could be particularly suitable for the early screening and identification of individuals unlikely to develop AD-related pathophysiology and for increasing the probability that individuals with AD pathophysiology are being selected for further investigations using more specific, expensive and/or more invasive methods with reduced accessibility such as PET imaging or CSF assessment. The broad availability of blood-based biomarkers will facilitate a critical step towards a cost-, resource- and time-effective multi-step diagnostic work-up and accelerate the re-engineering of drug Research & Development (R&D) pipelines, from proof of pharmacology to clinical trial design.

The plasma Aβ1-42/Aβ1-40 ratio performs well in predicting the presence of brain amyloid as assessed through amyloid-PET across the AD clinical continuum. Moreover, reduced plasma Aβ1-42/Aβ1-40 is significantly associated with the overall increased risk for developing AD [336]. For measures of Aβ1-42/Aβ1-40 in blood, a variety of techniques have been employed, including single-molecule arrays (SiMoA) technology [337–339], immunoprecipitation coupled with mass spectrometry (IP-MS) [340], IP coupled with liquid chromatography-mass spectrometry [341], immune-magnetic reduction [342], and stable isotope labeling kinetics protocols [343, 344]. While these analytical methods provide similar positive results in MCI, dementia, and cognitively healthy individuals at risk for AD, IP-MS methods have higher concordance with brain amyloidosis than Simoa assays [344]. Some of the approaches, however, are not scalable and/or have high variability. Hence, like CSF, fully automated assays offer a viable solution for blood-based biomarkers of brain Aβ accumulation [345, 346].

### Aβ oligomers in bodily fluids

With accumulating evidence of soluble Aβ oligomers and protofibrils being the critical toxic species within the Aβ pathway, the accurate detection and quantification of these species could prove useful for diagnostic and therapeutic context-of-use [166]. Different technologies are used for their detection/measurement, including enzyme-linked immunosorbent assay (ELISA), flow cytometry, nanoscale optical biosensors, amplified plasmonic exosome (APEX), single particle detection, nanoparticle-based detection, single-molecule fluorescence microscopy, a protein misfolding cyclic amplification assay method, and an assay based on the monoclonal antibody BAN50 [344, 347–351]. Most of these studies show a trend toward high CSF levels of Aβ oligomers in AD patients compared with healthy controls, but have proven controversial and, in some cases, with no clear discrimination between the groups, especially when involving prodromal AD. In many studies, oligomer concentrations were higher in MCI than AD or cognitively healthy individuals; there was significant overlap among concentrations in different populations [318, 352]. In some studies, patients with MCI who later converted to AD had increased Aβ oligomers CSF concentrations on a group level, but several samples had undetectable levels [353].

Assays to measure Aβ oligomers in plasma are under development [354–358]. A recently developed ELISA-Multimer Detection System (MDS), capable of differentiating multimers from their cellular monomers, detected higher plasma Aβ oligomers concentrations in AD versus healthy controls (HC) [359]. Before adapting MDS in clinical settings, further studies are needed to validate plasma Aβ oligomer concentration and use of the assay for screening patients, monitoring longitudinal changes across the course of AD, or determining the efficacy of Aβ-targeting drugs.

A study has been performed in human bodily fluids to assesses whether AD patients have higher levels of protofibrils compared with cognitively healthy controls. An enzyme-linked immunospot (ELISpot)-based investigation reported that AD patients display a significantly higher number of cells producing antibodies toward Aβ42 protofibrils compared to healthy controls [190]. Although the study did not directly assess plasma levels of protofibrils, it showed there is a specific immune response to the toxic Aβ protofibrils, which is significantly increased in AD patients [190].

### PET radioligands of brain amyloid plaques

**Fig. 6:**
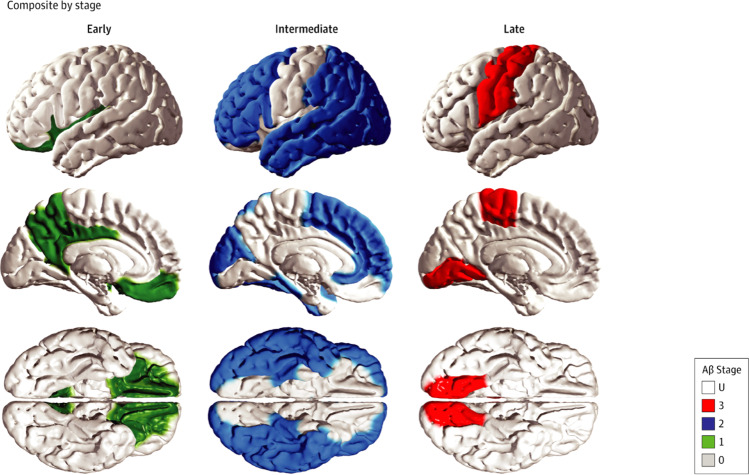
Techniques of in vivo Alzheimer’s disease amyloid-β pathway staging, along the clinical continuum, based on molecular imaging and innovative algorithms. Neocortical distribution of [^18^F]-florbetapir is shown in a composite representation according to Aβ stages. Early composite (positive in stage 1 in green; left), intermediate composite (positive in stage 2 in blue; middle) and late composite (positive in stage 3 in red; right) can allow global and regional assessment of amyloid plaque deposition. Adapted with permission from: ref. [393].

Molecular imaging in amyloid in AD is characterized by radiopharmaceuticals binding to aggregated insoluble fibrillary forms of cortical Aβ visualized via PET (Aβ-PET) [360]. Aβ neuroimaging allows (1) in vivo assessment of global and regional deposition of amyloid plaques, (2) exploration of the spatiotemporal relationships between brain Aβ accumulation (see Fig. 6), other pathomechanistic alterations of AD, and clinical outcomes, and (3) assessment of target engagement and treatment effect in anti-Aβ clinical trials as a quantifiable biomarker [361]. The FDA label for PET imaging emphasizes that a low Aβ-PET burden is incompatible with AD as the cause of the cognitive decline. Most older cognitively unimpaired or MCI individuals with low Aβ-PET burden will not develop or progress to AD in their lifetime [362]. Such a recommendation highlights the importance of employing a panel of biomarkers along with PET as prognostic indicators.

The first validated radiopharmaceutical developed for Aβ-PET was [^11^C]-PiB, a derivative of the amyloid-binding fluorescent dye thioflavin-T, which is a small molecule known to bind amyloid proteins aggregated into a cross β structure [363, 364]. [^11^C]-PiB has a short half-life, which limits its use to clinical centers with an on-site cyclotron and specialized radiochemistry expertise. Recently, the FDA has approved fluorine-18 [^18^F]-labeled compounds—[^18^F]-Florbetapir ([^18^F]-AV-45, AmyvidTM), [^18^F]-Florbetaben ([^18^F]-FBB, NeuraceqTM), [^18^F]-Flutemetamol ([^18^F]-FMT, VizamylTM)—that have a 110-min half-life, thus allowing for centralized production and regional distribution [365, 366].

Multi-center studies, systematic reviews, and meta-analyses of the PET radiotracers demonstrate substantial corroborating data for the capability Aβ-PET to differentiate AD patients from healthy controls (HC) and to predict the likelihood of progression to AD dementia in patients with MCI [367–369]. The results for sensitivity range from 89 to 97% by all study subgroups (HC versus AD dementia and HC versus MCI versus AD dementia individuals). The values ranged more widely, from 63% to 93%, for specificity [367–369]. Unlike neuroimaging of neurodegeneration and tau pathophysiology, the pattern of Aβ-PET deposition across the AD clinical spectrum (typical and atypical variants) does not show much regional differences [370].

Amyloid-PET imaging is primarily approved to be used as a binary visual reading approach (ordinal classification of positive or negative scans) to distinguish individuals with no/sparse Aβ plaques from those with moderate-to-frequent plaques. Recently, automatized pipelines that allow standardized quantitative measures have been developed. Quantitative studies enable regional investigation of brain Aβ deposition, allowing for tracking spatiotemporal evolution throughout the AD clinical continuum [13, 371]. These findings demonstrate a predictable regional sequence that may be used to stage an individual’s progress of in vivo cerebral amyloid pathology [371]. Regional Aβ staging based on amyloid-PET imaging has the potential to predict progression to cognitive impairment and dementia in individuals with preclinical and prodromal AD, with the most advanced amyloid stages able to identify high-risk groups of progression from MCI to dementia [371, 372]. For quantitative purposes, the three different tracers show considerable variability when measured using the typical SUVRs. To improve the comparability of the retention measurements across tracers and across centers, the Centiloid method has been proposed [360]. This method linearly scales the measure of a particular tracer from 0 to 100 scale, where “0” represents the average tracer retention in young controls, and “100” corresponds to the average racer retention in typical AD patients at the dementia stage [360].

Although radiopharmaceuticals target fibrillar Aβ, this does not represent a specific marker for a particular pool of Aβ, but for the global cerebral Aβ load [373]. For instance, AD patients with APP Arctic mutation or the Osaka variant show markedly low cerebral deposition of plaques as assessed through the Aβ-PET global SUVR [183, 200, 201]. On this basis, [^11^C]-PiB was tested for its ability to bind Aβ protofibrils and oligomers. Tritiated PiB ([^3^H]-PiB) bound strongly to Aβ1-42 fibrils and satisfactorily to protofibrils [374]. An earlier study also showed that PiB has three-fold less affinity for soluble forms than insoluble forms [375].

Concerning radiolabeled antibodies, recombinant antibody-based radioligand [^124^I]A3 could target soluble Aβ protofibrils [376, 377]. The radioligand di-scFv [^124^I]3D6-8D3 has a larger dynamic range and sensitivity for measuring more soluble forms of Aβ than [^11^C]-PiB [378]. Hence, antibody-based radioligands might visualize more subtle and earlier Aβ alterations than the conventional ones. There are several issues concerning this developing approach, including (i) technical difficulty to get antibodies into the brain in enough quantities to attain useful neuroimaging-based clinical information, (ii) the amount of soluble pools of Aβ is ~1% of all amount of Aβ in the brain and do not last long enough in the soluble form to allow imaging in the CNS, (iii) antibodies enter the brain very slowly, and maximal concentrations might take more than 1 day restricting its use to radiolabeling with very long T1/2 radioisotopes [379]. Future strategies to circumvent these physiological barriers include the use of nanoparticles, exosomes, or molecular chaperones that facilitate transport across the BBB.

## Conclusions

Over the recent decades, translational and multi-disciplinary studies—from (epi)genetic, to biological, and to biomarker-based clinical investigations—have contributed to unveiling the biochemical, physiological, and pathophysiological features of the Aβ pathway, including its spatial and temporal dynamics throughout the AD continuum. All point to the Aβ pathway as a hallmark of disease pathophysiology rather than a passive readout of the disease process. As discussed above, anatomical and biomarker-based studies of familial and sporadic AD provide critical genetic and molecular evidence about the initiation of the Aβ pathway decades before the onset of the symptoms and upstream to other pathophysiological hallmarks of AD.

These advances in biology have culminated in the identification of tangible therapeutic molecular targets for AD in order to slow disease progression at the earliest possible clinical and preclinical stages. Progress in drug R&D has also been accelerated by the validation of Aβ biomarkers-based outcomes and endpoints and for different context(s)-of-use, including patient diagnosis for clinical trials, target engagement of drug candidates, and proof-of-mechanism. Implementation of biomarker-guided pipelines contributes to explaining why the first generation of compounds targeting Aβ aggregation species and with putative disease-modifying effect reached late-stage development and exhibited phase II and phase III failures. However, the field needs to fully uncover the physiological functions of the Aβ pathway, as well as the upstream molecular orchestrators of its dyshomeostasis in AD. Aβ homeostasis undergoes a complex interplay consisting of highly conserved feedback loops and interactions among an array of quality control mechanisms and protein clearance pathways across cells, tissues, and body systems. Understanding this hierarchical organization across tissues and body systems and its decline with aging and in an individual, genetically determined fashion will be essential to comprehensively target the Aβ cycle for preventive strategies. New multi-modal imaging integrative approaches coupled with molecular imaging and fluid biomarkers hold the potential to unravel the spatial and temporal coordinates the Aβ pathways dynamics and to map the critical genetic and biological factors influencing sub-population clinical and pathophysiological trajectories.

Within this conceptual framework, Aβ-oriented therapies will be more and more scaled to the disease stage and biological inter-individual differences for time-sensitive and effective pathway (a mechanism)-based preventive strategies for AD, aligned with the precision medicine paradigm.
